# A Novel Anthropometry-Based Model to Estimate Appendicular Muscle Mass in Brazilian Older Women

**DOI:** 10.1155/jare/1053086

**Published:** 2025-03-29

**Authors:** Carlos Aiello Ribeiro, Lorena Rosa, Jorge Mota, Nádia Lima da Silva, Paulo Farinatti

**Affiliations:** ^1^Graduate Program in Exercise and Sport Sciences, Institute of Physical Education and Sports, University of Rio de Janeiro State, Rio de Janeiro, Brazil; ^2^Research Center in Physical Activity Health and Leisure (CIAFEL), Faculty of Sports, Laboratory for Integrative and Translational Research in Population Health (ITR), University of Porto, Porto, Portugal

**Keywords:** aging, appendicular skeletal muscle mass, isokinetic strength testing, muscle function

## Abstract

**Background:** The assessment of appendicular skeletal muscle mass (ASM) is central to the diagnosis of sarcopenia (SA). We developed an anthropometric model for estimating ASM and tested its validity to identify SA and associated risk of disability (RSA) in older women.

**Methods:** The equation was developed with 89 women (60–88 years, 72 ± 6 years), with a cross-validation sample of 12 women (60–84 years, 67 ± 5 years). Validity was determined through concordance between actual versus estimated ASMs, correlations between actual/estimated ASM versus peak torque (PT) and total work (TW) during isokinetic knee extension/flexion and handgrip strength, and agreement of patients classified with SA and RSA.

**Results:** The predictive equation was ASM (kg) = 0.177 (body mass, kg)–0.075 (arm circumference, cm) + 0.020 (thigh circumference, cm) + 5.376 (*R* = 0.905; *R*^2^ = 0.819; *R*^2^ad = 0.809; *F* = 86.96; *p* < 0.0001; SEE = 1.35 kg). Agreement between actual and estimated ASMs was confirmed by validation (ICC = 0.81; *p* < 0.0001) and cross-validation samples (ICC = 0.72, *p* < 0.035). Regression characteristics in PRESS statistics (*R*^2^ PRESS = 0.79; SEE-PRESS = 1.61) were compatible with the original model. Percent agreements for the classification of SA and RSA from indices calculated using actual/estimated ASM were 98% (gamma = 0.98, *p* = 0.015) and 68% (gamma = 0.89, *p* < 0.0001) in validation and 67% (gamma = 1.0, *p* = 0.032) and 70% (gamma = 0.84, *p* < 0.001) in cross-validation samples. Correlations between actual/estimated ASM versus PT (range 0.57–0.76, *p* < 0.05), TW (range 0.51–0.75, *p* < 0.05), and handgrip (range 0.67–0.74, *p* < 0.001) were theoretically consistent, being moderate and similar in both samples.

**Conclusion:** This new anthropometric model has satisfactory prediction qualities and could be applied as a simple and practical method for estimating ASM in Brazilian older women.

## 1. Introduction

Sarcopenia refers to a significant decline in skeletal muscle mass (SMM), strength, and functional ability, serving as an important indicator of frailty [1]. This condition commonly arises with aging and negatively affects the capacity to perform daily activities [1, 2]. Older adults frequently experience a loss of muscle strength linked to this decline, which can also lead to adverse metabolic effects, increasing the risk of chronic diseases like obesity and diabetes [3, 4].

The loss of muscle strength and mass plays a critical role in functional dependence among the elderly. Consequently, detecting muscle mass loss is vital for health interventions aimed at older populations [1, 5, 6]. Common assessment models employ indices based on appendicular skeletal muscle mass (ASM) and SMM, along with strength measurements [7, 8]. In assessing sarcopenia risk and potential disability, these indices are particularly relevant [1]. To classify individuals as sarcopenic, researchers often use sex-specific thresholds that are two standard deviations below average values found in younger populations [2, 9].

Gold-standard methods for measuring ASM include dual-energy X-ray absorptiometry (DXA), though access to these techniques can be limited, especially in larger studies and lower-income regions [10]. There is an ongoing need for simple, valid, and cost-effective methods to estimate SMM [11, 12]. Developing alternative ways to measure ASM and SMM indices could facilitate early sarcopenia diagnosis, monitor intervention effectiveness, and consider the functional aspects of strength performance [13]. It is essential for the criteria used to classify sarcopenia and associated disability risk to align with the actual muscle function.

Simple regression models can estimate ASM, aiding in the classification of individuals as sarcopenic or not. Since ASM encompasses the muscle mass of both upper and lower limbs, it may also be indirectly assessed using arm and leg perimeters. Various prediction models have been created to estimate the total SMM among older individuals, showing validity in their application [7, 12, 14–19]. Some equations specifically estimate ASM using appendicular perimeters [12, 14, 15], though many incorporate more complex variables that are not as easily obtainable [7, 16–19]. Additionally, several models have used highly correlated variables like body mass and body mass index (BMI) [20–22], or relied solely on body mass and height, which may lack specificity [16, 18, 22, 23].

Notably, there has been limited research on the applicability of these prediction equations across different populations, particularly due to ethnic variations in muscle mass [12, 24]. Moreover, previous studies indicate that predictive equations for ASM developed for mixed populations often do not apply adequately to women due to variations in muscle mass distribution and anthropometric and hormonal differences [9, 24]. These studies underscore the importance of developing predictive equations that consider gender-specific variations in muscle mass distribution and hormonal influences to ensure accurate assessments in women. Another relevant issue is the hormonal transition that occurs with female aging, especially during the postmenopausal period, when muscle loss intensifies due to reduced estrogen levels. This factor differentiates the progression of sarcopenia between women and men and may impact the applicability of equations developed for both sexes.

In Brazil, there are a few ASM prediction models [18, 20], with only one study incorporating forearm circumference as an independent variable [20]. Recent findings highlight the poor cross-validity of the existing equations for predicting ASM in Brazilian women [20, 25], suggesting that this issue may stem not only from gender specificity but also from the country's ethnic diversity, underscoring the need for equations tailored to this population.

Given the high costs of directly measuring ASM, developing practical diagnostic methods for the initial sarcopenia screening is crucial, especially in developing countries. Although using arm and leg perimeters to predict ASM is theoretically sound, models utilizing these measurements for older adults are scarce. Therefore, this study aims to introduce and validate a straightforward statistical model for estimating ASM (and consequently SMM) in older women. The validation process includes comparing the actual ASM with estimated values and exploring the relationship between ASM and strength in both validation and cross-validation samples, as well as testing the model's effectiveness as a quick tool for identifying potential sarcopenia and associated disability risks.

## 2. Methods

### 2.1. Sample

The study included 89 community-dwelling women aged 60–88 years. A subgroup of 12 participants, aged 60–84, was selected for cross-validation purposes. Participants were excluded based on the following criteria: (a) medical conditions preventing DXA assessments, (b) ongoing anabolic hormone therapy or use of ergogenic aids for muscle gain, and (c) cardiovascular, respiratory, bone, muscle, or joint issues that could hinder strength testing. This research is part of a larger clinical trial approved by the Ethics Review Board of the University of Rio de Janeiro State (CAAE: 20235319.7.0000.5259). All participants provided informed consent before their involvement in the study.

### 2.2. Experimental Design

Participants were recruited during two distinct periods via social media and advertisements in university facilities. Data collection took place over two laboratory visits, both in the morning to reduce circadian effects on strength performance (8:00–11:00 a.m.). During the initial visit, participants completed the informed consent process and underwent anthropometric and body composition assessments, including measurements of height, weight, arm and thigh circumferences, and DXA. The second visit focused on measuring the isokinetic strength of knee extensors and flexors. Variables included in the predictive model were sex, body weight, BMI, body fat percentage, and arm and thigh circumferences.

Two criteria were established to validate the predictive model: the ASM measured via DXA should align with the estimated values from our equation, and classifications of participants as sarcopenic or at high risk of physical disability should match those determined by Baumgartner's and Janssen's cutoff points, respectively. Furthermore, the actual and estimated ASM and SMM should correlate with strength performance in both validation and cross-validation groups, as sarcopenia is fundamentally defined by the loss of muscle mass, strength, and functionality [1, 26].

### 2.3. Procedures

#### 2.3.1. Anthropometric Measurements

Body weight and height were measured using digital scales (Filizola, São Paulo, SP, Brazil) and a stadiometer (Sanny, São Bernardo do Campo, SP, Brazil). BMI was calculated (kg/m^2^). Arm circumference was recorded at the midpoint of the dominant arm during a muscle contraction (elbow flexed at 90°). Thigh circumference was measured at the midpoint on the lateral side of the dominant leg. Measurements were taken with a flexible metal tape (Sanny, American Medical, São Bernardo do Campo, SP, Brazil).

#### 2.3.2. Isokinetic and Handgrip Strengths

Lower limb strength was assessed using an isokinetic dynamometer (Biodex System 4 PRO, Biodex Medical Systems, Inc., Shirley, NY, USA). Participants began with a 5-minute warm-up on a cycle ergometer (Cateye EC-1600, Cateye, Tokyo, Japan) followed by familiarization with the dynamometer through 15 repetitions at 120°/s. The strength testing involved three sets of 10 repetitions for knee extension and flexion, with 120-s rest intervals, using the dominant limb. The range of motion was set from 0° to 90° at a speed of 60°/s. Peak torque (Nm) and total work (J) were recorded, with validity confirmed if the coefficient of variation (CV) between sets was less than 15%. Handgrip strength was measured in the dominant hand using a dynamometer (Grip A, Tokyo, Japan) following the American Society of Hand Therapists [27] guidelines. Participants performed three submaximal contractions for warm-up, followed by three maximal contractions separated by 60-s rest intervals, with the highest value recorded.

#### 2.3.3. Assessment of Body Composition and Appendicular Muscle Mass

Body composition was analyzed via DXA (Lunar iDXA, GE Healthcare, Chalfont St. Giles, United Kingdom) using the enCORE software. This analysis provided regional and total body metrics, including body weight, lean mass, fat mass, and bone mineral density. Although DXA may underestimate sarcopenia due to challenges in distinguishing lean mass from other tissues, it remains the gold standard for body composition assessment. Lean mass in the arms and legs (kg) was utilized to calculate ASM and the ASM index (ASM [kg]/height [m^2^]) [7]. Additionally, SMM was estimated from ASM using the equation proposed by Janssen et al. [8] (SMM = [1.17 × ASM]–1.01), and SMM was normalized for height to derive the skeletal muscle index (SMM index).

#### 2.3.4. Classification of Sarcopenia and Risk for Physical Disability

ASM index cutoff points [7] were employed to identify participants as sarcopenic (5.45 kg/m^2^), established as two standard deviations below sex-specific means from reference data in the Rosetta Study of younger adults (ages 18–40). For females, SMM index cutoffs of 5.76–6.75 and ≤ 5.75 kg/m^2^ were used to indicate moderate and high risks for physical disability, respectively [8].

### 2.4. Statistical Analyses

**Figure 1:**
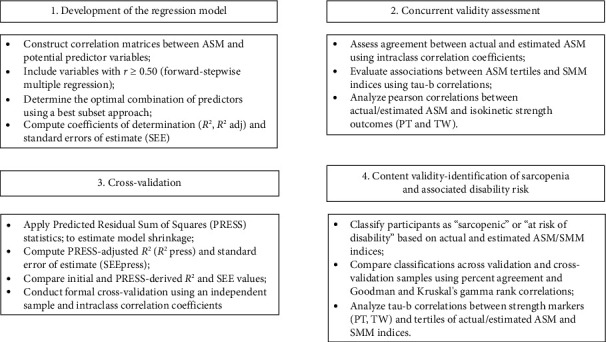
Flowchart of data analysis.

Data normality was confirmed using the Shapiro–Wilk test, and results are expressed as mean ± standard deviation. Analyses were performed using Statistica 10.0 software (Statsoft, Tulsa, OK, USA), with a significance level set at *p* ≤ 0.05. We detail procedures adopted by our group in a previous study that validated a model to estimate ASM in another population [13].  Figure 1 summarizes the combined approaches used to conduct this study.

#### 2.4.1. Development of the Regression Model

Correlation matrices were constructed between ASM and potential predictor variables, including only those with moderate or higher correlations (*r* ≥ 0.50) in a forward-stepwise multiple regression procedure to create a predictive equation for ASM. The optimal combination of independent variables was determined using a best subset approach, and coefficients of determination (*R*^2^, *R*^2^adj) along with standard errors of estimate (SEE) were calculated.

#### 2.4.2. Concurrent Validity Assessment

The predictive model's concurrent validity was evaluated by assessing the agreement between actual and estimated ASMs through intraclass correlation coefficients. The association between tertiles of ASM and SMM indices was verified using tau-b correlations, alongside Pearson correlations between estimated and actual ASMs versus isokinetic strength outcomes—peak torque (PT) and total work (TW) during knee flexion/extension.

#### 2.4.3. Cross-Validation

Cross-validation of the predictive equations was performed using Predicted Residual Sum of Squares statistics (PRESS) to account for potential model shrinkage when applied to independent yet comparable samples. PRESS employs a jackknife method that repeatedly refits the model, excluding one observation each time to predict and calculate residuals for that observation [28]. The PRESS adjusted *R*^2^ (*R*^2^ press) was computed, and the PRESS standard error of estimate (SEEpress) was calculated. These metrics were compared with the initial *R*^2^ and SEE. Additionally, formal cross-validation was conducted with an independent sample to confirm findings obtained via PRESS statistics, using intraclass correlation coefficients to compare assessed versus estimated ASMs [13].

#### 2.4.4. Content Validity—Identification of Sarcopenia and Associated Disability Risk

Participants were classified as “sarcopenic” or “at risk of disability due to sarcopenia” based on actual and estimated ASM and SMM indices, as defined by Baumgartner's and Janssen's cutoff points. This classification was compared between validation and cross-validation samples through percent agreement and Goodman and Kruskal's gamma rank correlations. Tau-b correlations were also utilized to examine the relationship between tertiles of isokinetic strength markers (PT, TW) and tertiles of actual and estimated ASM and SMM indices.

## 3. Results

Table 1 depicts the characteristics of body composition, isokinetic, and handgrip strength in validation and cross-validation samples. Overall, individuals were eutrophic or overweight. No difference was found between validation and cross-validation samples.

Table 2 presents Pearson coefficients between ASM determined by DXA and potential predictors. Correlations > 0.5 were detected for body mass, arm circumference, and thigh circumference, and these variables were retained for inclusion in a multiple-stepwise regression model with ASM as the dependent variable. The predictive equation issued from this model was ASM (kg) = 0.177 (body mass, kg) − 0.075 (arm circumference, cm) + 0.020 (thigh circumference, cm) + 5.376 (*R* = 0.905; *R*^2^ = 0.819; *R*^2^adj = 0.809; *F* = 86.96; *p* < 0.0001; SEE = 1.35 kg). Values obtained for PRESS statistics (*R*^2^ PRESS = 0.79 and SEE-PRESS = 1.61 kg) were compatible with data from the regression model.

**Figure 2:**
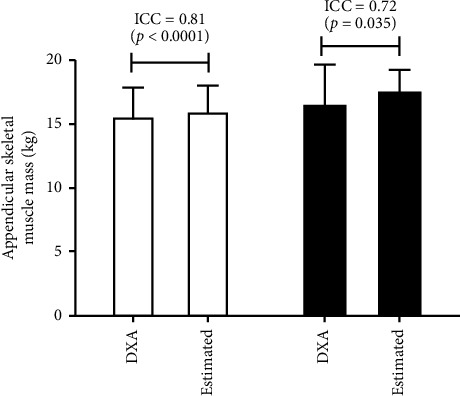
Appendicular skeletal muscle mass obtained through DXA and estimated using the present model in validation (*n* = 89) and cross-validation samples (*n* = 12). DXA, dual X-ray absorptiometry; ICC, intraclass correlation coefficient.

Figure 2 shows that strong and similar intraclass correlation coefficients were found between actual and estimated ASMs in validation (Panel 1A) and cross-validation (Panel 1B) samples.

Table 3 presents the percent agreement and Goodman and Kruskal's gamma rank correlations between actual and estimated ASM and SMM indices. The fairness of our model was ratified by a moderate-to-good agreement of tertiles (validation sample) and median values (cross-validation sample) for both indices. The small relative variation across ranks of actual and estimated ASM and SMM indices seemed not to affect the classification of “sarcopenia” and “risk of disability due to sarcopenia.” In the validation sample, the overall percent agreement for the classification of “sarcopenia” based on actual and estimated ASM indices was 98% (two discrepancies; gamma = 0.98, *p*=0.015). In the cross-validation sample, the agreement was 67% (four discrepancies; gamma = 1.0, *p*=0.032). Noteworthy, only six participants were identified with sarcopenia in the validation sample (approximately 10%) and four participants in the cross-validation sample (approximately 30%). In the validation sample, the identification of sarcopenia based on our model overestimated one case and underestimated one case versus DXA. In the cross-validation sample, the classification was underestimated in four individuals.

The model accurately classified 68% of participants in the validation sample as being “at risk of disability due to sarcopenia” based on the cutoff points for the SMM index (25 discrepancies, gamma = 0.89; *p* < 0.0001) and 70% of participants in the cross-validation sample (four discrepancies, gamma = 0.84, *p* < 0.001). In the validation sample, the “risk of disability” was overestimated in three instances and underestimated in 22 cases. Extreme misclassification—where the risk was wrongly placed into the lowest or highest category—occurred only once (as an underestimation). In the cross-validation sample, overestimation occurred in one case, while underestimation was observed in three cases.

Table 4 presents correlations between actual and estimated ASMs versus strength markers. The moderate to strong associations in validation and cross-validation samples were equivalent and theoretically consistent with age-related decreases in muscle mass related to lower muscle strength.

Table 5 reinforces these data, presenting tau-b correlations between ranks of actual and estimated ASM and SMM indices versus isokinetic (PT, TW) and handgrip strength. The correlations were similar in both validation and cross-validation samples. Albeit coefficients have been low to moderate, they generally reflected consistent and positive associations between muscle mass and strength performance.

## 4. Discussion

This study proposed a model for estimating ASM, designed to provide a quick and straightforward method for classifying older women as “sarcopenic” or “nonsarcopenic.” The validation included comparing actual and estimated ASMs, assessing ASM and SMM indices in validation and cross-validation samples, and calculating correlation coefficients versus strength outcomes. Our results indicate that the predictive model is valid and could serve as a useful tool for identifying sarcopenia and related disability risks in this specific group.

Age-related muscle mass reduction is acknowledged as a factor contributing to disability and increased mortality in older adults [1, 6]. Sarcopenia is a complex condition involving the interplay between muscle mass and function [1], making early detection and intervention crucial for maintaining independence and quality of life [11]. However, current methods for assessing muscle loss are often costly and require specialized training, highlighting the need for accessible alternatives that offer insights into sarcopenia risk through indirect assessments.

The theoretical basis for using arm and thigh circumferences in predictive models is supported by evidence that skinfold-circumference models demonstrate higher accuracy in predicting muscle mass compared to those based on body mass and height [11, 14]. The previous research has showed strong correlations between various circumferences and ASM—our model aligns with these findings [13, 15], and its validity was tested using three criteria: (a) the precision of ASM estimation, (b) the identification of “sarcopenic” individuals using estimated ASM based on Baumgartner's cutoff points, and (c) the assessment of those at “risk of disability due to sarcopenia” using estimated SMM calculated indirectly from ASM through Janssen's cutoff points.

Our model demonstrated a high correlation between actual and estimated ASMs, explaining over 85% of the variance in both validation and cross-validation samples. This robust correlation extended to muscle function as measured by isokinetic and handgrip strengths. Regarding the second aspect, there was a strong agreement in identifying “sarcopenic” versus “nonsarcopenic” individuals based on both actual and estimated ASMs, with approximately 80% agreement and a rank correlation of 0.86 between the two indices. The relatively low proportion of sarcopenic individuals in the validation sample (10% versus 30% in cross-validation) may have influenced these results but aligns with reported prevalence rates in community-dwelling older adults [7, 29].

While previous models developed for older adults integrated various independent variables—body mass, height, and/or BMI [16, 18, 22, 23], arm skinfolds [16, 18, 20], handgrip strength [7, 19], corrected muscle area [16], or ethnicity [21]—our approach focused on simple measurements. Although some existing models have shown good predictive properties, their application in identifying sarcopenia and associated disability risks remains unexplored. For this reason, the effectiveness of our model was evaluated for both estimating ASM and classifying the risk of disability due to sarcopenia. Overall, the agreement in risk classifications based on the SMM index was less robust than that for identifying sarcopenic individuals, with around 70% agreement observed in validation and cross-validation samples and moderate-to-strong gamma correlations. This discrepancy may result from the specific nature of the SMM index, which involves indirect calculations that may introduce variability in populations with differing muscle losses and functions [8].

The concurrent validity analysis indicated that the cutoff points proposed by Baumgartner and Janssen effectively identified “sarcopenia” and “risk of disability,” correlating with variations in muscle functions measured through strength performance. This is clinically significant as muscle strength deficits are a primary consequence of age-related muscle loss [1, 4]. Additionally, it is essential to integrate muscle mass and function over time for accurate sarcopenia diagnosis, which should not rely solely on visual assessments of muscle mass [30].

## 5. Strengths and Limitations of the Study

Our model proved to be sensitive to variations in muscle function, with lower strength associated with poorer estimated ASM, thereby increasing the likelihood of sarcopenia classification. Given its simplicity, this ASM estimation model could be instrumental in monitoring functional impairment risks in practical intervention settings. While DXA remains the gold standard for body composition analysis [10], its accessibility is limited due to cost and the need for specialized training. The present model offers an efficient and affordable alternative for estimating ASM using straightforward anthropometric measures, making it applicable in community-based settings to track interventions to mitigate sarcopenia.

Limitations of this study include a relatively small sample size and the homogeneity of participants, all of whom were functionally independent community dwellers. This exclusion of individuals with advanced sarcopenia or frailty suggests that our model may be better suited for early detection and prevention in less compromised individuals, thereby enhancing its clinical validity. Future research should assess the model's validity in larger, more diverse populations. Including only women may also be considered a limitation to the external validity of our results. However, beyond physiological reasons, selecting a homogeneous group probably reduced data variability and improved statistical analysis precision, allowing for a more robust model applicable to older women.

## 6. Conclusion

The present study presents a novel, cost-effective model for estimating ASM in older women, based on easily obtainable data: ASM (kg) = 0.177 (body mass, kg) − 0.075 (arm circumference, cm) + 0.020 (thigh circumference, cm) + 5.376. Actual and estimated ASM values were closely aligned, demonstrating strong correlation, with the model accounting for over 85% of variance as assessed by DXA. These results support the model's practical utility for the initial screening of sarcopenia and associated disability risks among Brazilian older women. Validation through both procedures confirms its relevance in primary care and exercise interventions aimed at preventing functional impairments related to age-associated muscle loss, warranting further prospective studies to solidify these findings.

## Figures and Tables

**Table 1:** Participants' clinical conditions, body composition, circumferences, and strength in validation and cross-validation samples (mean ± standard deviation).

Variables	Validation sample (*n* = 89)	Cross-validation sample (*n* = 12)
*Subject characteristics*		
Age (years)	71.8 ± 6.1	67.2 ± 5.4
Height (cm)	154.2 ± 6.5	159.4 ± 7.1
Body mass (kg)	64.1 ± 11.4	62.2 ± 9.1
Body mass index (kg/m^2^)	26.9 ± 4.0	28.4 ± 3.4
Total fat (%)	42.1 ± 6.7	45.1 ± 3.7
Arm circumference (cm)	30.5 ± 3.5	32.8 ± 3.7
Thigh circumference (cm)	41.8 ± 6.4	54.8 ± 5.9
ASM (kg)	15.3 ± 2.3	16.3 ± 3.3
SMM (kg)	16.8 ± 2.7	18.1 ± 3.8
ASM index (kg/m^2^)	6.4 ± 0.8	6.4 ± 1.1
SMM index (kg/m^2^)	7.1 ± 0.9	7.1 ± 1.2

*Isokinetic and handgrip strengths*		
Peak torque EXT (Nm)	81.8 ± 23.3	89.9 ± 29.2
Peak torque FLX (Nm)	37.7 ± 10.4	44.2 ± 16.6
Total work EXT (J)	743.9 ± 183.2	788.5 ± 261.4
Total work FLX (J)	347.2 ± 115.5	396.1 ± 164.8
Handgrip (kg)	22.1 ± 4.6	20.2 ± 5.6

**Table 2:** Pearson/Spearman correlations between appendicular muscle mass (ASM) and potential predictive variables (*n* = 89).

Variables	ASM (kg)	*p* value
Body mass (kg)	0.803	< 0.0001
Body mass index (kg/m^2^)	0.482	0.004
Arm circumference (cm)	0.581	< 0.0001
Thigh circumference (cm)	0.501	< 0.0001
Body fat (%)	−0.099	0.378

**Table 3:** Percent agreement of individuals with ASM and SMM indices classified in the same rank as defined by tertiles (validation sample) or median (cross-validation sample) and gamma correlations between overall ranks.

Validation sample (*n* = 89)	1st tertile (%)	2nd tertile (%)	3rd tertile (%)	Total (%)	Gamma
ASM index	85.2	70.1	78.6	78.1	0.86⁣^∗^
SMM index	81.5	70.4	75.9	75.6	

**Cross-validation sample (*n* = 12)**	**< Median (%)**	**> Median (%)**	—	**Total (%)**	**Gamma**

ASM index	80.0	80.0		80.0	0.88⁣^∗^
SMM index	80.0	80.0		80.0	

*Note:* ASM = appendicular skeletal muscle mass, SMM = total skeletal muscle mass.

⁣^∗^: *p* < 0.001.

**Table 4:** Pearson correlations between actual and estimated appendicular muscle mass (ASM) versus isokinetic outcomes during knee extension/flexion and handgrip strength in validation and cross-validation samples.

	**Validation sample (*n* = 89)**	
	**Actual ASM**	**Estimated ASM**

Peak torque (extension)	0.71⁣^∗∗∗^	0.69⁣^∗∗∗^
Peak torque (flexion)	0.57⁣^∗∗∗^	0.60⁣^∗∗∗^
Total work (extension)	0.76⁣^∗∗∗^	0.75⁣^∗∗∗^
Total work (flexion)	0.51⁣^∗∗∗^	0.58⁣^∗∗∗^
Handgrip strength	0.67⁣^∗∗∗^	0.68⁣^∗∗∗^

	**Cross-validation sample (*n* = 12)**	
	**Actual ASM**	**Estimated ASM**

Peak torque (extension)	0.76⁣^∗∗∗^	0.69⁣^∗∗^
Peak torque (flexion)	0.73⁣^∗∗∗^	0.58⁣^∗^
Total work (extension)	0.76⁣^∗∗∗^	0.66⁣^∗∗^
Total work (flexion)	0.56⁣^∗^	0.51⁣^∗^
Handgrip strength	0.74⁣^∗∗∗^	0.72⁣^∗∗∗^

⁣^∗^: *p* < 0.05.

⁣^∗∗^: *p* < 0.01.

⁣^∗∗∗^: *p* < 0.001.

**Table 5:** Tau-b correlations between ranks of isokinetic strength outcomes during knee extension and flexion versus ASM and SMM indices in validation (tertile ranks) and cross-validation (median ranks) samples.

	PT extension	PT flexion	TW extension	TW flexion	Handgrip
*Validation (n = 89)*					
ASM index_actual_	0.47⁣^∗∗∗^	0.46⁣^∗∗∗^	0.42⁣^∗∗∗^	0.31⁣^∗^	0.39⁣^∗∗^
ASM index_estimated_	0.39⁣^∗∗∗^	0.37⁣^∗∗∗^	0.36⁣^∗∗∗^	0.33⁣^∗∗^	0.31⁣^∗∗^
SMM index_actual_	0.46⁣^∗∗∗^	0.47⁣^∗∗∗^	0.42⁣^∗∗∗^	0.31⁣^∗^	0.30⁣^∗∗^
SMM index_estimated_	0.37⁣^∗∗∗^	0.38⁣^∗∗∗^	0.32⁣^∗∗∗^	0.32⁣^∗∗^	0.31⁣^∗∗^

*Cross-validation (n = 12).*					
ASM index_actual_	0.78⁣^∗∗∗^	0.67⁣^∗∗∗^	0.60⁣^∗∗^	0.67⁣^∗∗∗^	0.28
ASM index_estimated_	0.60⁣^∗∗^	0.58⁣^∗^	0.42⁣^∗^	0.56⁣^∗^	0.40⁣^∗^
SMM index_actual_	0.80⁣^∗∗∗^	0.67⁣^∗∗^	0.60⁣^∗∗^	0.67⁣^∗∗^	0.29
SMM index_estimated_	0.60⁣^∗∗^	0.48⁣^∗^	0.40	0.48⁣^∗^	0.28

*Note:* ASM, appendicular skeletal muscle mass; SMM, total skeletal muscle mass.

Abbreviations: PT, peak torque; TW, total work.

⁣^∗^: *p* < 0.05.

⁣^∗∗^: *p* < 0.01.

⁣^∗∗∗^: *p* < 0.001.

## Data Availability

The data supporting the findings of this study are available from the corresponding author upon reasonable request.

## References

[1] Cruz-Jentoft A. J., Baeyens J. P., Bauer J. M. (2010). Sarcopenia: European Consensus on Definition and Diagnosis. *Age and Ageing*.

[2] Denison H. J., Cooper C., Sayer A. A., Robinson S. M. (2015). Prevention and Optimal Management of Sarcopenia: A Review of Combined Exercise and Nutrition Interventions to Improve Muscle Outcomes in Older People. *Clinical Interventions in Aging*.

[3] Curcio F., Ferro G., Basile C. (2016). Biomarkers in Sarcopenia: A Multifactorial Approach. *Experimental Gerontology*.

[4] Fragala M. S., Cadore E. L., Dorgo S. (2019). Resistance Training for Older Adults: Position Statement From the National Strength and Conditioning Association. *The Journal of Strength & Conditioning Research*.

[5] Doherty T. J. (2003). Invited Review: Aging and Sarcopenia. *Journal of Applied Physiology*.

[6] Lauretani F., Russo C. R., Bandinelli S. (2003). Age-Associated Changes in Skeletal Muscles and Their Effect on Mobility: An Operational Diagnosis of Sarcopenia. *Journal of Applied Physiology*.

[7] Baumgartner R. N., Koehler K. M., Gallagher D. (1998). Epidemiology of Sarcopenia Among the Elderly in New Mexico. *American Journal of Epidemiology*.

[8] Janssen I., Baumgartner R. N., Ross R., Rosenberg I. H., Roubenoff R. (2004). Skeletal Muscle Cutpoints Associated With Elevated Physical Disability Risk in Older Men and Women. *American Journal of Epidemiology*.

[9] Gallagher D., Visser M., De Meersman R. E. (1997). Appendicular Skeletal Muscle Mass: Effects of Age, Gender, and Ethnicity. *Journal of Applied Physiology*.

[10] Guglielmi G., Ponti F., Agostini M., Amadori M., Battista G., Bazzocchi A. (2016). The Role of DXA in Sarcopenia. *Aging Clinical and Experimental Research*.

[11] Lustgarten M. S., Fielding R. A. (2011). Assessment of Analytical Methods Used to Measure Changes in Body Composition in the Elderly and Recommendations for Their Use in Phase II Clinical Trials. *The Journal of Nutrition, Health & Aging*.

[12] Wen X., Wang M., Jiang C. M., Zhang Y. M. (2011). Anthropometric Equation for Estimation of Appendicular Skeletal Muscle Mass in Chinese Adults. *Asia Pacific Journal of Clinical Nutrition*.

[13] Farinatti P., Paes L., Harris E. A., Lopes G. O., Borges J. P. (2017). A Simple Model to Identify Risk of Sarcopenia and Physical Disability in HIV-Infected Patients. *The Journal of Strength & Conditioning Research*.

[14] Lee R. C., Wang Z., Heo M., Ross R., Janssen I., Heymsfield S. B. (2000). Total-Body Skeletal Muscle Mass: Development and Cross-Validation of Anthropometric Prediction Models. *The American Journal of Clinical Nutrition*.

[15] Martin A. D., Spenst L. F., Drinkwater D. T., Clarys J. P. (1990). Anthropometric Estimation of Muscle Mass in Men. *Medicine & Science in Sports & Exercise*.

[16] Kulkarni B., Kuper H., Taylor A. (2013). Development and Validation of Anthropometric Prediction Equations for Estimation of Lean Body Mass and Appendicular Lean Soft Tissue in Indian Men and Women. *Journal of Applied Physiology*.

[17] Ramirez E., Enríquez-Reyna M. C., Garza-Sepúlveda G., Tijerina-Sáenz A., Ramos-Peña E., de la Garza M. G. (2015). Puntos de corte y Validación de una Ecuación Antropométrica Para Estimar la Masa Muscular, en el Estudio de la Sarcopenia en Población Mexicana. *Salud Publica de Mexico*.

[18] Gomes I. C., Gobbo L. A., Silva A. M. (2013). Appendicular Lean Soft Tissue: Development and Cross-Validation of Predictive Models for Older Men and Women. *The Journal of Frailty & Aging*.

[19] Lera L., Albala C., Angel B. (2014). [Anthropometric Model for the Prediction of Appendicular Skeletal Muscle Mass in Chilean Older Adults]. *Nutricion Hospitalaria*.

[20] Pereira P. M., da Silva G. A., Santos G. M., Petroski E. L., Geraldes A. A. (2013). Development and Validation of Anthropometric Equations to Estimate Appendicular Muscle Mass in Elderly Women. *Nutrition Journal*.

[21] Santos L. P., Gonzalez M. C., Orlandi S. P. (2019). New Prediction Equations to Estimate Appendicular Skeletal Muscle Mass Using Calf Circumference: Results From NHANES 1999-2006. *Journal of Parenteral and Enteral Nutrition*.

[22] Visvanathan R., Yu S., Field J. (2012). Appendicular Skeletal Muscle Mass: Development and Validation of Anthropometric Prediction Equations. *The Journal of Frailty & Aging*.

[23] Tanko L. B., Movsesyan L., Mouritzen U., Christiansen C., Svendsen O. L. (2002). Appendicular Lean Tissue Mass and the Prevalence of Sarcopenia Among Healthy Women. *Metabolism*.

[24] Silva A. M., Shen W., Heo M. (2010). Ethnicity-Related Skeletal Muscle Differences Across the Lifespan. *American Journal of Human Biology*.

[25] Abdalla P. P., Bohn L. S., Santos A. P. (2022). Cross-Validation of 20 Anthropometric Prediction Equations for Appendicular Muscle Mass in Older Brazilian Women: A Cross-Sectional Study. *Geriatr Gerontol Aging*.

[26] Marty E., Liu Y., Samuel A., Or O., Lane J. (2017). A Review of Sarcopenia: Enhancing Awareness of an Increasingly Prevalent Disease. *Bone*.

[27] MacDermid J., Solomon G., Valdes K. (2015). *ASHT Clinical Assessment Recommendations*.

[28] Holiday D. B., Ballard J. E., McKeown B. C. (1995). PRESS-Related Statistics: Regression Tools for Cross-Validation and Case Diagnostics. *Medicine & Science in Sports & Exercise*.

[29] Rolland Y., Lauwers-Cances V., Cournot M. (2003). Sarcopenia, Calf Circumference, and Physical Function of Elderly Women: A Cross-Sectional Study. *Journal of the American Geriatrics Society*.

[30] Burton L. A., Sumukadas D. (2010). Optimal Management of Sarcopenia. *Clinical Interventions in Aging*.

