# Correction to “A Novel Anthropometry‐Based Model to Estimate Appendicular Muscle Mass in Brazilian Older Women”

**DOI:** 10.1155/jare/9898035

**Published:** 2025-12-22

C. A. Ribeiro, L. Rosa, J. Mota, N. L. da Silva, and P. Farinatti, “A Novel Anthropometry‐Based Model to Estimate Appendicular Muscle Mass in Brazilian Older Women,” *Journal of Aging Research* 2025, no. 1 (2025): 1–9, https://doi.org/10.1155/jare/1053086.

In the article titled “A Novel Anthropometry‐Based Model to Estimate Appendicular Muscle Mass in Brazilian Older Women,” some information in the Funding section was omitted.

The funding statement should read as follows:

“This study was funded in part by the Coordination for the Improvement of Higher Education Personnel—Brazil (CAPES)—Finance Code 001 and grants from the Carlos Chagas Filho Foundation for Research Support in the State of Rio de Janeiro (FAPERJ, E‐26/202.880/2017, recipient P.F.), National Council for Technological and Scientific Development (CNPq, 303629/2019‐3, recipient P.F.), and Foundation for Science and Technology (UIDB/00617/2020, doi: 10.54499/UIDB/00617/2020; UIDP/00617/2020, doi: 10.54499/UIDP/00617/2020, recipient J.M.).”

We apologize for this error.

